# Advanced small extracellular vesicles delivery systems for *in situ* tissue engineering

**DOI:** 10.20517/evcna.2025.149

**Published:** 2026-03-13

**Authors:** Yike Gao, Jingyi Sang, Zhuo Wan, Yue Wang, Xiaojing Yuan, Yuqing Dong, Zuoying Yuan, Yuming Zhao

**Affiliations:** ^1^Department of Pediatric Dentistry, Peking University School and Hospital of Stomatology, National Engineering Laboratory for Digital and Material Technology of Stomatology, and Beijing Key Laboratory of Digital Stomatology, Beijing 100081, China.; ^2^Center of Basic Medical Research, Institute of Medical Innovation and Research, Peking University Third Hospital, Beijing 100191, China.

**Keywords:** *In situ* tissue engineering, sEVs, controlled delivery, hydrogels, surface modification

## Abstract

*In situ* tissue engineering, which activates the body’s innate regenerative capacity, has demonstrated superior clinical translation potential than traditional *ex situ* approaches. Small extracellular vesicles (sEVs), as natural nanovesicles, can excellently mimic the paracrine functions of cells and are thus emerging as promising cell-free alternatives for *in situ* tissue engineering. Despite advantages such as low immunogenicity, multi-target regulatory capabilities, and cross biological barriers availability, the therapeutic sustainability of sEVs is limited by their rapid clearance *in vivo*, underscoring the need for effective delivery systems. This review systematically summarizes the sources and bioactivities of sEVs, delineates the design principles and technological advances in sEVs delivery systems, and highlights their application in tissue engineering, while also outlining future trajectories for the development of intelligent delivery platforms.

## INTRODUCTION

Conventional tissue engineering strategies often involve the transplantation of *in vitro* pre-constructed grafts consisting of scaffolds, cells and growth factors^[1]^. However, such approaches face certain limitations and risks inherent to *ex vivo* cell manipulation, including donor site morbidity, immune rejection, poor cell homing and engraftment efficacy, tumorigenic potential, and ethical/regulatory constraints^[2]^. As an innovative strategy that bypasses the need for *ex vivo* cell manipulation, *in situ* tissue engineering aims to directly mobilize the host’s innate regenerative capacity by implanting bioactive materials that guide the functional reconstruction of injured tissues^[3,4]^. This process requires the recruitment of endogenous cells to the injury site and the induction of tissue regeneration, either by modulating the extracellular microenvironment or by directing cellular reprogramming^[5]^.

Small extracellular vesicles (sEVs) are vesicles with diameters between 30-150 nm secreted by cells, carrying abundant lipids, nucleic acids [DNA, RNA, microRNA (miRNA)], and proteins^[6]^. By delivering biomolecules to recipient cells, sEVs mediate intercellular communication and regulate key physiological processes, including immune responses, angiogenesis, and tissue regeneration^[7]^. Owing to their low immunogenicity, multi-target regulatory capabilities, and ability to cross biological barriers, sEVs have been regarded as ideal substitutes for exogenous cells in tissue engineering^[8-11]^.

**Figure 1:**
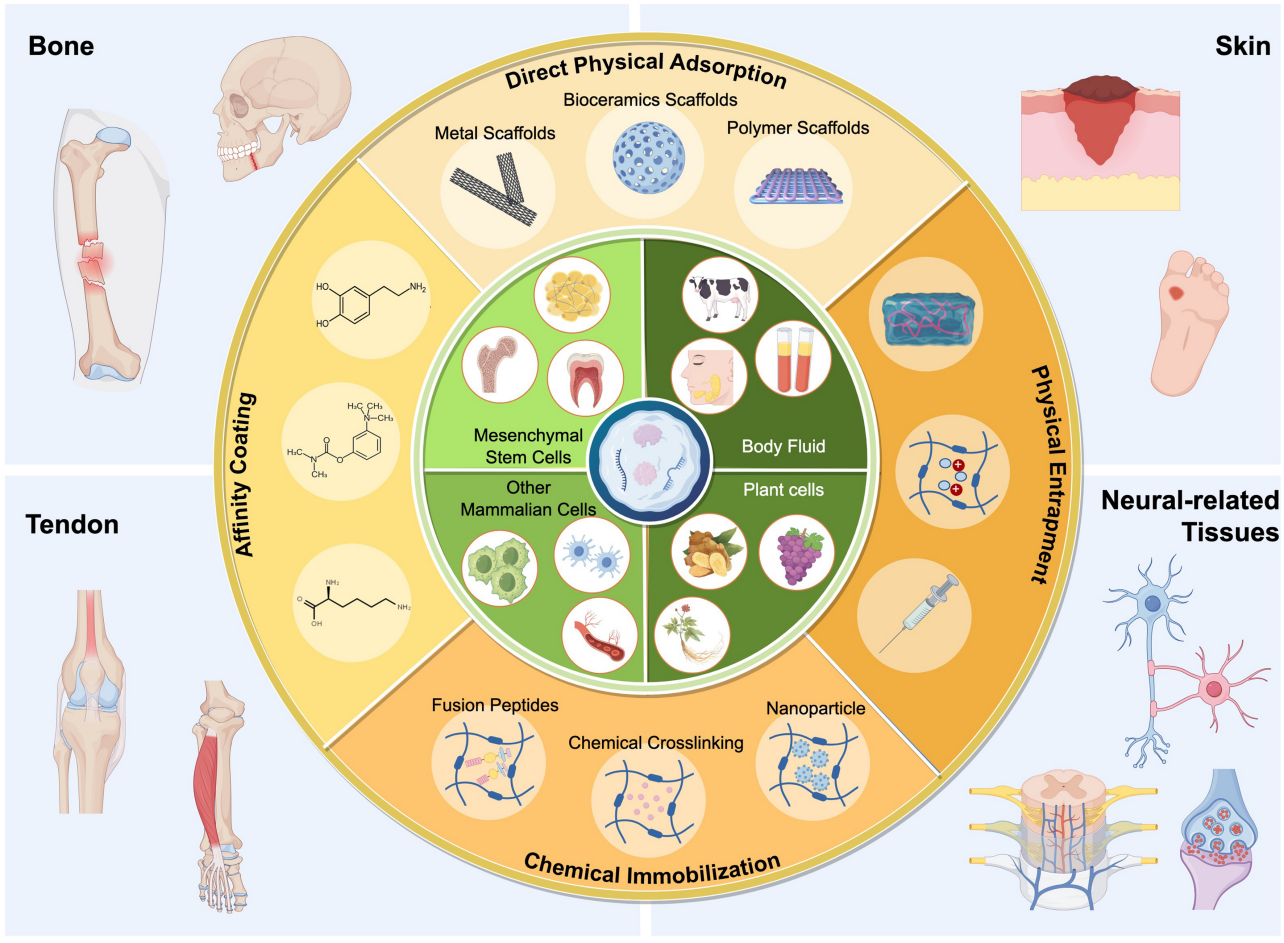
Current status and innovations of sEVs delivery systems for *in situ* tissue engineering (The schematic illustration was created using FigDraw). sEVs: Small extracellular vesicles.

The therapeutic efficacy of sEVs is dose-dependent^[12]^. However, when administered via local or systemic injection, a significant portion of sEVs often accumulates in off-target organs (e.g., liver, spleen, and kidneys) and undergoes rapid clearance. This limits the sustainability of their therapeutic effects and often necessitates repeated administrations. Biomaterial-based strategies, grounded in the principles of *in situ* tissue engineering, offer a promising platform to overcome these delivery challenges by enabling spatiotemporally controlled release of sEVs^[13,14]^. These biomaterial-based systems are designed not only to prolong therapeutic durability via sustained release but also to improve targeting precision. More importantly, the finely tuned biophysical and biochemical properties of these biomaterials can further modulate the regenerative microenvironment, which holds profound implications for *in situ* tissue regeneration^[15,16]^. Here, we systematically review the cutting-edge advances in engineered sEVs delivery systems for *in situ* tissue engineering, including sEVs sources and bioactivities, delivery systems design, applications, and future directions [Figure 1].

## SEVs

The unique properties of sEVs, including their ability to transport bioactive cargo reflective of donor cell states, position them as key mediators in intercellular communication and promising tools for addressing central challenges in *in situ* tissue regeneration^[17]^. Their functional capabilities are diverse and source-dependent, enabling therapeutic effects such as immunomodulation, promotion of cell proliferation/migration, and stimulation of angiogenesis^[18,19]^. The key features of biological functions of natural and functionalized sEVs from different sources are compared in Table 1.

**Table 1:** Biological functions of natural and functionalized sEVs from different sources

**Type**	**Sources**	**Functionalized strategy**	**Biological functions**	**Ref.**
Mammalian cell-derived sEVs	BMSCs	Natural	Promotes BMSC proliferation, induces macrophage polarization towards the M2 phenotype, and facilitates bone regeneration	[20]
		sEVs membrane surface modification: receptor-ligand interactions	Mediates bone-targeted delivery and accumulation of sEVs (sEVs) via GP1, thereby mitigating bone loss through the promotion of osteoblast-mediated bone formation	[21]
	ADSCs	Natural	Alleviates inflammation, promotes angiogenesis, collagen deposition, cell proliferation, and migration, thereby accelerating wound healing	[22]
		Content modification of sEVs: lentiviral transfection	Improves the dermal cell growth cycle and promotes dermal cell autophagy to alleviate senescence in damaged dermal cells, thereby stimulating hair regeneration	[23]
		Content modification of sEVs: hypoxia treatment	Promotes the migration, proliferation, and angiogenic potential of HUVECs, accelerating diabetic wound healing	[24]
	DPSCs	Natural	Promotes the polarization of macrophages towards the M2 phenotype, alleviating alveolar bone resorption	[25]
		Content modification of sEVs: odontogenic differentiation induction	sEVs derived from preconditioned cells exhibit enhanced effects in inducing the proliferation, migration, and tube formation of HUVECs, facilitating the regeneration of the pulp-dentin complex	[26]
	Macrophage	Natural	M2 macrophage-derived sEVs promote macrophage polarization to the M2 phenotype, protecting myocardial tissue	[27]
		Content modification of sEVs: ultrasound-loaded medications	Macrophage-derived sEVs are utilized to target microglia in the central nervous system, delivering resveratrol to alleviate multiple sclerosis	[28]
	HUVECs	Natural	In co-delivery with Tazarotene, it promotes cell proliferation, migration, and angiogenesis, accelerating diabetic wound healing	[29]
		Content modification of sEVs: 3D culture	*In vitro*, it promotes the proliferation and migration of fibroblasts	[30]
	Neural progenitor cells	sEVs membrane surface modification: receptor-ligand interactions	Following intravenous administration via the tail vein, it targets the ischemic brain region, exerting anti-inflammatory effects and suppressing post-stroke inflammation	[31]
Body fluid-derived sEVs	Platelet-rich plasma	Natural	Promotes macrophage polarization to the M2 phenotype and stimulates neovascularization	[32]
	Milk	Natural	Serves as a delivery vehicle for insulin to treat type 1 diabetes	[33]
Plant cells release sEV-like nanoparticles	Ginseng	Natural	Promotes the polarization of macrophages from the M2 to the M1 phenotype, inhibiting melanoma growth	[34]
	Ginger	Natural	Induces Nrf2 nuclear translocation in hepatocytes, suppressing oxidative damage and preventing alcohol-induced liver injury	[35]
	Portulaca oleracea L	Natural	Induces the expansion of CD4+CD8+ T cells for the treatment of inflammatory bowel disease	[36]
	Sesame leaves	Content modification of sEV: luteolin-encapsulated	Compared to sEVs derived from sesame leaves and free luteolin, luteolin-encapsulated sEVs exhibit superior antioxidant and anti-inflammatory efficacy	[37]

sEVs: Small extracellular vesicles; BMSCs: bone marrow-derived mesenchymal stem cells; ADSCs: adipose-derived stem cells; DPSCs: dental pulp stem cells; HUVECs: human umbilical vein endothelial cells; GP1: Golgi glycoprotein 1; M2: M2 macrophage phenotype; CD4+CD8+: CD4-positive and CD8-positive T cells; Nrf2: nuclear factor erythroid 2-related factor 2.

### Mesenchymal stem cell-derived sEVs

Mesenchymal stem cells (MSCs), including bone marrow mesenchymal stem cells (BMSCs), adipose-derived stem cells (ADSCs), dental pulp stem cells (DPSCs), and stem cells from human exfoliated deciduous teeth (SHEDs), possess self-renewal and multi-lineage differentiation capabilities. MSC-derived sEVs (MSC-sEVs) are among the most used sEVs, exhibiting functions similar to MSCs^[38]^.

BMSCs have low immunogenicity, are easily isolated from bone marrow, and exhibit strong self-renewal and multi-lineage differentiation potential (e.g., osteoblasts, chondrocytes, neural cells). As donor cells for sEVs, they show significant advantages in regenerative medicine and disease treatment. Wang *et al*. demonstrated that BMSC-derived sEVs significantly enhanced BMSC osteogenic differentiation *in vitro* and induced bone regeneration in a mouse calvarial defect model^[18]^. Guo *et al*. showed that bone-targeted delivery of BMSCs-derived sEVs alleviated bone loss in colitis mice, promoted bone formation, and accelerated fracture healing^[21]^. Li *et al*. found that BMSCs-derived sEVs promoted rotator cuff healing by polarizing macrophages from pro-inflammatory M1 to anti-inflammatory M2 phenotypes, reducing post-surgical inflammatory responses^[39]^. In spinal cord injury repair, BMSCs-derived sEVs exhibited immunomodulatory and tissue-regenerative effects: regulating microglial polarization toward M2, synergistically enhancing neuronal and oligodendrocyte differentiation of neural stem cells, inhibiting astrocyte differentiation, and promoting axon growth^[40]^.

Adipose tissue, as the largest endocrine organ regulating metabolism and the immune system, is widely distributed in the human body. ADSCs, isolated from adipose tissue, offer advantages including minimal invasiveness, high extraction yield, and fewer ethical controversies^[41]^. Their derived sEVs have thus become emerging platforms for cell-free tissue repair and regenerative medicine. Tao *et al*. found ADSC-derived sEVs can enhance cell proliferation, promote angiogenesis, and encourage macrophage polarization toward the M2 phenotype, thereby mitigating inflammatory responses and promoting tissue repair^[15]^. In another study, coupled with a decellularized extracellular matrix, sustained-release ADSC-derived sEVs showed efficacy in treating intervertebral disc degeneration through regulating matrix metalloproteinases to balance matrix synthesis/degradation and by inhibiting pyroptosis and through alleviating inflammation^[42]^. In COVID-19, ADSC-derived sEVs significantly alleviated lung injury in patients^[43]^.

Additionally, DPSCs, sourced from dental pulp tissue, are easily accessible and exhibit anti-inflammatory, immunomodulatory, and soft/hard tissue-inducing potential. DPSC-derived sEVs have been extensively applied in dental pulp regeneration, bone regeneration, and immune regulation^[26,44-46]^. Shen *et al*. showed that DPSC-derived sEVs delivered miR-1246 to promote M2 macrophage polarization, modulate immune responses, and reduce bone resorption in periodontitis mice^[25]^. Intravenous injection of DPSC-derived sEVs suppressed neuroinflammation and microglial pyroptosis in subarachnoid hemorrhage via the miR-197-3p/FOXO3 axis^[47]^. SHEDs, a special DPSC type obtained non-invasively from exfoliated deciduous teeth with minimal ethical concerns, exhibit stronger cell migration and vascularization capabilities than DPSCs^[48]^. In LPS-induced wound healing, SHED-derived sEVs promoted wound closure and reduced itching by inducing macrophage migration and enhancing autophagy^[49]^. Another study found that SHED-derived sEVs inhibited microglial activation via the mitogen-activated protein kinase (MAPK) pathway, alleviating trigeminal neuralgia^[50]^. In our previous work, we extracted sEVs from hypoxia-preconditioned SHEDs and validated their enhanced vascularization potential via the vascular endothelial growth factor (VEGF) signal pathway^[51]^.

### Other mammalian cell-derived sEVs

Immune cells, as sEV donors, offer unique advantages in tissue regeneration, particularly in targeting inflammatory or tumor sites and innate immunomodulatory capabilities^[52-54]^. Immune cell-derived sEVs may carry donor-derived integrins, chemokine receptors, *etc*., enabling active homing to injury sites^[55]^. For instance, macrophage-derived sEVs can target central nervous system microglia, delivering resveratrol to alleviate multiple sclerosis^[28]^. Concurrently, immune cell-derived sEVs carry immunomodulatory molecules (e.g., cytokines, chemokines, miRNA) to precisely regulate local inflammation, creating favorable microenvironments for regeneration^[56]^. Hsu *et al*. recently found that CD55 on neutrophil-derived vesicles inhibits complement C3 convertase activation, exerting stable anti-inflammatory effects and reducing neutrophil recruitment/tissue damage^[57]^. M2 macrophage-derived sEVs enriched with lncRNA AK083884 regulated macrophage metabolic reprogramming to alleviate myocardial inflammation and dysfunction in mice^[27]^. Liu *et al*. found that monocyte-derived sEVs deliver miR-223 to endothelial cells, where it directly targets signal transducer and activator of transcription 3 (STAT3), suppresses its phosphorylation, and consequently downregulates the expression of interleukin 1 beta (IL-1β), interleukin 6 (IL-6), vascular cell adhesion protein 1 (VCAM-1), and intercellular adhesion molecule 1 (ICAM-1), thereby contributing to the attenuation of vascular inflammation^[58]^. Neutrophil-derived sEVs carrying miR-30d-5p inhibit suppressor of cytokine signaling 1 (SOCS-1) and NAD-dependent protein deacetylase sirtuin-1 (SIRT1) in macrophages, which leads to activation of the nuclear factor kappa-light-chain-enhancer of activated B cells (NF-κB) pathway, upregulation of NLR family pyrin domain containing 3 (NLRP3) inflammasome expression, promotion of M1 polarization, and initiation of pyroptosis in the context of sepsis-related acute lung injury^[59]^.

Angiogenesis is fundamental to tissue repair, and mismatched vascularization can lead to abnormal healing in skin, neural, or bone tissues. Endothelial cells participate in vasculogenesis. Their derived sEVs may possess enhanced angiogenic potential. Human umbilical vein endothelial cell (HUVEC)-derived sEVs combined with tazarotene significantly accelerated cell proliferation, migration, and angiogenesis both *in vitro* and *in vivo*, facilitating diabetic wound repair^[29]^. Neural progenitor cells (NPCs), derived from midbrain regions, maintain normal morphology/differentiation after prolonged passaging (> 45 passages). Tian *et al*. designed a recombinant protein-modified sEV derived from NPCs. This engineered sEV delivers specific miRNAs, such as let-7g-5p and miR-99a-5p, which inhibit the MAPK pathway by reducing p38 phosphorylation. Consequently, the expression of pro-inflammatory cytokines [tumor necrosis factor alpha (TNF-α), IL-1β, and IL-6] is downregulated, leading to attenuated neuroinflammation and demonstrating stable anti-inflammatory effects in a mouse cerebral ischemia model^[31]^. Additionally, sEVs isolated from cardiomyocytes may play key roles in mediating pathological fibrotic remodeling, promoting cardiac angiogenesis, and inhibiting myocardial infarction^[60-62]^.

### Body fluid-derived sEVs

Body fluids, particularly blood and breast milk, represent scalable sources of therapeutic sEVs that possess intrinsic regenerative properties and the capability to cross biological barriers. Platelet-derived sEVs exhibit potential anti-inflammatory and pro-angiogenic activities, effectively promoting diabetic wound healing. For instance, sEVs delivered *via* a dissolvable microneedle system stimulated endothelial cell tube formation *in vitro*, induced anti-inflammatory M2 macrophage polarization through bioactive cargo (e.g., platelet-derived growth factor, transforming growth factor-beta, and specific microRNAs), and accelerated wound closure in diabetic mice^[32]^. Distinct from the wound healing context, reticulocyte-derived blood sEVs exhibit natural brain-targeting ability via transferrin-receptor interactions; dopamine-loaded versions aided Parkinson’s disease treatment^[63]^.

Milk-derived sEVs (e.g., from bovine milk), present a promising platform for drug delivery due to their excellent biocompatibility, consistent availability, and scalable production. Proof-of-concept studies have demonstrated their potential in this domain. For example, paclitaxel-loaded milk sEVs achieved significant systemic efficacy *in vivo*, overcoming the inherent solubility and absorption challenges associated with oral paclitaxel administration^[64]^. Nevertheless, the precise mechanisms governing their gastrointestinal translocation remain an active area of research. In models of type 1 diabetes, insulin was successfully loaded into milk sEVs using sonication to overcome the lipid bilayer barrier. The resulting oral formulation achieved more significant and sustained hypoglycemic effects than subcutaneous insulin injections^[33]^. Notably, loading methodology affects the efficiency of hydrophilic drug encapsulation. A comparative study confirmed that active loading techniques such as sonication and freeze-thaw cycles significantly outperformed passive incubation in retaining hydrophilic drugs^[65]^.

### Plant-derived extracellular vesicle-like nanoparticles

Although mammalian cell-derived sEVs show regenerative effects, challenges include long extraction cycles and low yields. Researchers have turned to fresh plant tissues as scalable and cost-effective alternatives. Plant cells release extracellular vesicle-like nanoparticles (PELNs), which are typically lipid bilayer vesicles with diameters ranging from 50 to 200 nm, sharing morphological similarities with mammalian sEVs^[36,66,67]^. In terms of composition, PELNs are enriched with specific phospholipids (e.g., phosphatidic acid, phosphatidylcholine), functional proteins (e.g., heat shock proteins, transmembrane transporters), and bioactive metabolites (e.g., polyphenols, flavonoids) from their source plants. Importantly, they also carry plant-specific small RNAs and miRNAs, which may contribute to cross-kingdom regulatory functions^[36,68-70]^. PELNs from ginger, ginseng, purslane, and grapefruit exhibit anti-inflammatory, antioxidant, antitumor, and gut microbiota-modulating effects, widely used for inflammatory bowel disease, alcohol-induced liver injury, cerebral ischemia, and melanoma^[34-36,68,71,72]^.

Gao *et al*. extracted PELNs from turmeric to treat ulcerative colitis. After oral administration, PELNs accumulated in inflamed colons and showed excellent anti-inflammatory activity in acute and chronic colitis models. Flow cytometry revealed that turmeric PELNs promoted M1-to-M2 macrophage polarization and restored damaged intestinal barriers^[73]^. In the context of central nervous system (CNS) disorders, Panax notoginseng-derived PELNs entered the brain and attenuated injury. Mechanistically, upon internalization by microglia, these PELNs promote a phenotypic switch from the pro-inflammatory M1 state to the anti-inflammatory M2 state, primarily via activation of the phosphoinositide 3-kinase/protein kinase B (PI3K/AKT) pathway. This reprogramming dampens neuroinflammation, which in turn reduces neuronal apoptosis, limits infarct volume, and helps maintain blood-brain barrier integrity^[74]^. Despite these advantages, PELNs lack the sophisticated innate surface ligands found on mammalian sEVs, potentially resulting in lower or less specific targeting. The precise targeting mechanism of plant-derived sEVs in humans remains a future research direction. Overall, plant-derived PELNs offer safe and cost-effective therapeutic options.

### Engineering strategies and molecular mechanisms for potentiating sEV efficacy

While the intrinsic properties of sEVs from various sources lay the foundation for their therapeutic potential, a deeper understanding of their mechanisms of action and the development of engineering strategies are key to advancing their clinical translation. Two central aspects warrant further discussion: the mechanisms governing sEVs targeting and the engineered pathways through which they exert precise biological control.

The targeting specificity of sEVs is largely dictated by their surface composition. Mammalian cell-derived sEVs inherit a complex array of membrane proteins from their parent cells. This molecular signature enables active, ligand-receptor-mediated targeting. For instance, stem cells from apical papilla (SCAP)-derived sEVs possess cellular characteristics similar to their parent SCAP cells. Cell division cycle 42 (Cdc42) deliverd by sEVs can be transferred to and reutilized by recipient endothelial cells, thereby promoting cell migration and facilitating angiogenesis^[75]^. In contrast, PELNs possess a distinct lipid and protein corona derived from the plant plasma membrane and cytosol^[76]^. This fundamental difference in origin dictates divergent biodistribution profiles and application paradigms.

To overcome limitations of natural sEVs, engineering strategies are employed to augment their function. Modifying the sEVs surface with targeting peptides [e.g., for CD63 molecule (CD63), arginylglycylaspartic acid (RGD) for integrins] directly dictates which cell the sEVs engage^[77,78]^. Research by Sun *et al*. demonstrated that conjugating umbilical cord MSCs-derived sEVs with a chondrocyte-affinity peptide significantly enhanced their chondrocyte-targeting ability and improved their potential for cartilage repair^[79]^. The core therapeutic effect frequently stems from the engineered cargo delivered into the cytoplasm of recipient cells. Regarding immunoregulation, engineered sEVs can carry miRNAs or proteins to achieve specific functions such as promoting osteogenesis or wound healing. For example, Ma *et al*. found that engineered sEVs enriched with miR-423-5p enhanced the development of secreted frizzled related protein 2 (Sfrp2)-positive osteogenic fibroblasts at periodontal defect sites relative to unmodified sEVs, leading to accelerated early osteogenesis and the regeneration of a native-like cementum-periodontal ligament (PDL)-alveolar bone complex^[80]^. In another study, Wei *et al*. demonstrated that sEVs engineered with miR-17-5p could target and inhibit senescence-associated factors phosphatase and tensin homolog (PTEN) and p21, thereby activating the PI3K/AKT signaling pathway in recipient cells. This activation countered cellular senescence by downregulating p16, p53, and reactive oxygen species (ROS) levels, while promoting angiogenesis and collagen synthesis via upregulation of hypoxia-inducible factor 1-alpha (HIF-1α), growth factors, and type I/III collagen, thereby synergistically restoring damaged tissues^[81]^.

## SCAFFOLD-BASED sEVS DELIVERY SYSTEMS

SEVs are rapidly cleared *in vivo* by the mononuclear phagocyte system, limiting local accumulation and duration of action^[82]^. Biomaterial-based delivery systems are crucial to overcome this limitation. These systems can be broadly classified by their core material form and integration mechanism. To clarify the classification framework used in this review, we distinguish between solid scaffolds and hydrogels. Scaffolds are defined here as solid or semi-solid, often porous, 3D structures that provide mechanical support and a framework for tissue ingrowth, with sEVs typically integrated onto their surfaces or into surface coatings. In contrast, hydrogels (discussed in Section HYDROGEL ENCAPSULATION) are highly hydrophilic, swollen 3D networks that encapsulate sEVs within their matrix.

Across both scaffolds and hydrogels, sEVs can be associated with the material via three primary functional strategies: direct Incorporation, affinity immobilization, and surface engineered targeting. The following sections detail the application of these strategies within scaffold-based systems and hydrogel systems.

Scaffold materials with 3D porous structures provide biophysical and biochemical cues for cell attachment, proliferation, and differentiation, while also serving as platforms for sEV delivery. They can be fabricated from natural polymers (e.g., collagen, chitosan), synthetic polymers [e.g., polylactic acid (PLA), poly(lactic-co-glycolic acid) (PLGA)], ceramics [e.g., β-tricalcium phosphate (β-TCP), hydroxyapatite], metals (e.g., titanium), and composites. The key features of scaffold-based systems are compared in Table 2.

**Table 2:** Comparison of surface coating methods to deliver EVs

**Material**	**Surface coating**	**Interaction**	**Source**	**EVs loading**	**Loading kinetics**	**Release kinetics**	**Application**	**Ref.**
Porous β-TCP	-	Physical adsorption	SHED-derived sEVs	100 μg sEVs/5 mg β-TCP	Not mentioned	50%-60% for 1 day, ~100% for 8 day	Alveolar bone defect repair	[83]
Collagen	-	Integrins binding	ADSCs-derived sEVs	200 μg/μL sEVs solution	Not mentioned	24% ± 4% in 1 day, 89% ± 3.6% in 21 day	Tissue regeneration and inflammatory regulation	[16]
PLGA scaffold	PDA	Mussel-inspired Adsorption	hADSCs-derived sEVs	1 μg/μL sEVs solution (250 μL/scaffold)	PLGA: 73.6 ± 22.4 μg/scaffold; PLGA/pDA: 165.72 ± 15.4 μg/scaffold	PLGA: 60% in 1 day, 100% in 4 day; pDA/PLGA: 20% in 1 day, ~80% in 8 day	Bone regeneration	[84]
PCL fibrous membranes	PEI	Electrostatic interactions	MSC-derived sEVs	2-45 μg/mL sEVs solution	< 30 μg/mL: loading efficiency ~100%; maximum loading amounts: ~35 μg/membranes	No sEVs release from scaffolds after the residual sEVs dissociation at a 2% level	Tissue-reparative immunomodulation	[85]
PCL scaffold	CaSi	Electrostatic interactions	MSC-derived sEVs	100 μg/mL sEVs solution	Not mentioned	PCL: 80% in 1 day, 100% in 5 day; pDA-PCL: 18% in 1 day, 30% in 20 day	Bone regeneration	[86]
PLGA scaffold	Double layered PDA	Mussel-inspired Adsorption and layer by layer coating	Smoothened agonist sterosome	Immobilization 0.1 or 1 mg/mL sterosome for 0.5-25 h	Immobilization 1 mg/mL sterosome: for 1 h: 13.0 ± 0.3 mg per scaffold	Without 2nd PDA: ~60% in 1 day, ~75% in 4 day, ~100% in 14 day With 2nd PDA: ~50% in 1 day, ~60% in 4 day, ~80% in 14 day	Bone regeneration	[87]
Porous polyetheretherketone (PEEK)	Fe^3+^ - TA	Hydrogen bond (polyphenol groups)	BMSC-derived sEVs	127.39 μg/cm^2^	Not mentioned	SPEEK: 90% in 3 day; TA-SPPEK: ~50% in 3 day, ~90% in 14 day	Promote osseointegration	[88]
Electrospun PCL vascular grafts	Heparin	Electrostatic interaction	MSC-derived sEVs	100 μg/mL for 4 h	0.59 ± 0.06 μg/mm^2^	Not mentioned	Inhibiting calcification of synthetic vascular grafts by immunomodulation	[89]
Porous PLGA microspheres	PDA	Mussel-inspired Adsoprtion	Hypoxia pretreated SHED-derived sEVs	1,000 μg/mL for 1-6 h	14.6 ± 2.6 μg/mg microspheres for 4 h	9.6% in 3 day, 48.3% in 7 day, 82.4% in 14 day, 90.2% in 21 day	Vascularized bone regeneration	[51]

β-TCP: Beta-tricalcium phosphate; SHED: stem cells from human exfoliated deciduous teeth; sEVs: small extracellular vesicles; ADSCs: adipose-derived stem cells; PLGA: poly(lactic-co-glycolic acid); PDA: polydopamine; hADSCs: human adipose-derived stem cells; PCL: polycaprolactone; MSC: mesenchymal stem cells; CaSi: calcium silicate; PEEK: polyetheretherketone; Fe^3+^: ferric ion; TA: tannic acid.

### Direct physical adsorption

**Figure 2:**
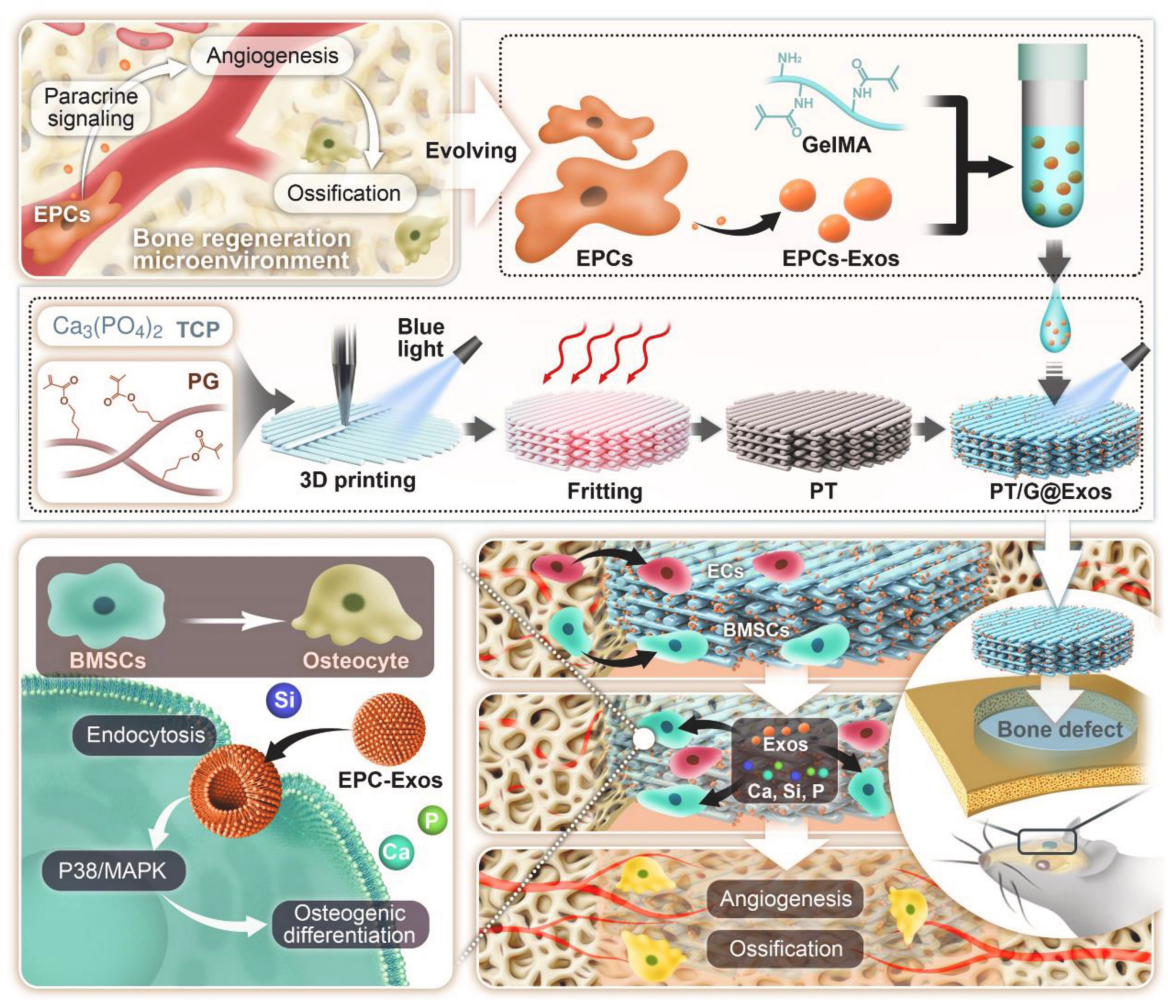
Schematic of the PT/G@Exos scaffold, where EPC-exos are encapsulated in GelMA and directly anchored onto the 3D-printed bioactive glass scaffold. The PG precursor binds TCP powder to form a 3D-printed PT scaffold. This design enables the sustained release of sEVs alongside Ca, Si, and P ions, thus orchestrating the angiogenesis-osteogenesis coupling to accelerate vascularized bone regeneration. Reprint with permission from Elsevier^[94]^. PT: 45S5 bioactive glass precursor scaffold; PG: photocurable 45S5 bioactive glass; Exos: exosomes; EPC-exos: endothelial progenitor cell-derived exosomes; sEVs: small extracellular vesicles; GelMA: gelatin methacryloyl; TCP: tricalcium phosphate.

As a direct incorporation strategy, physical adsorption is the simplest method for integrating sEVs into scaffolds, achieved by dripping sEV suspension onto materials or immersing scaffolds in sEV solutions. 3D-printed PLA scaffolds adsorbed with human gingival tissue-derived sEVs promoted bone regeneration^[90]^. Ying *et al*. designed β-TCP scaffolds delivering BMSC-sEVs, observing neovascularization and bone regeneration in calvarial defects^[91]^. β-TCP adsorbed with human induced pluripotent stem cell-derived MSC-sEVs released 60% of sEVs within 24 h and fully released in ~5 days. sEV/β-TCP scaffolds significantly enhanced BMSC proliferation, migration, and osteogenesis *in vitro*, with high osteocalcin (OCN) expression and osteogenic activity in rat calvarial defect^[92]^. Hydroxyapatite scaffolds adsorbed with BMSC-sEVs enhanced bone formation *in vitro* and improved osteoinduction via sustained release^[93]^. Kong *et al*. incorporated endothelial progenitor cell-derived sEVs into gelatin methacryloyl (GelMA) hydrogel, which was then anchored onto a 3D-printed 45S5 bioactive glass/tricalcium phosphate scaffold. This composite system accelerated bone regeneration in rat defects by coupling angiogenesis with osteogenesis [Figure 2]^[94]^.

Metal scaffolds (e.g., titanium alloys) offer superior mechanical strength for bone repair. Zhai *et al*. constructed 3D-printed titanium scaffolds delivering MSC-sEVs. Adsorption efficiency peaked at 79.48% after 12-h co-incubation (higher than 24 h). sEVs were continuously released, with 50% released within 2 h^[95]^. Wu *et al*. mixed Schwann cell-derived sEVs into Matrigel hydrogel injected into porous titanium scaffolds, validating efficacy in rabbit femoral condyle defects^[96]^. Graphene coatings enhance stem cell osteogenesis^[97,98]^. Sun *et al*. used graphene-modified porous titanium scaffolds delivering ADSC-sEVs to repair rabbit mandibular defects^[99]^. Direct physical adsorption is simple but relies on passive diffusion, often causing uncontrolled burst release. Thus, scaffold modifications are needed to improve delivery precision.

### Affinity coating

**Figure 3:**
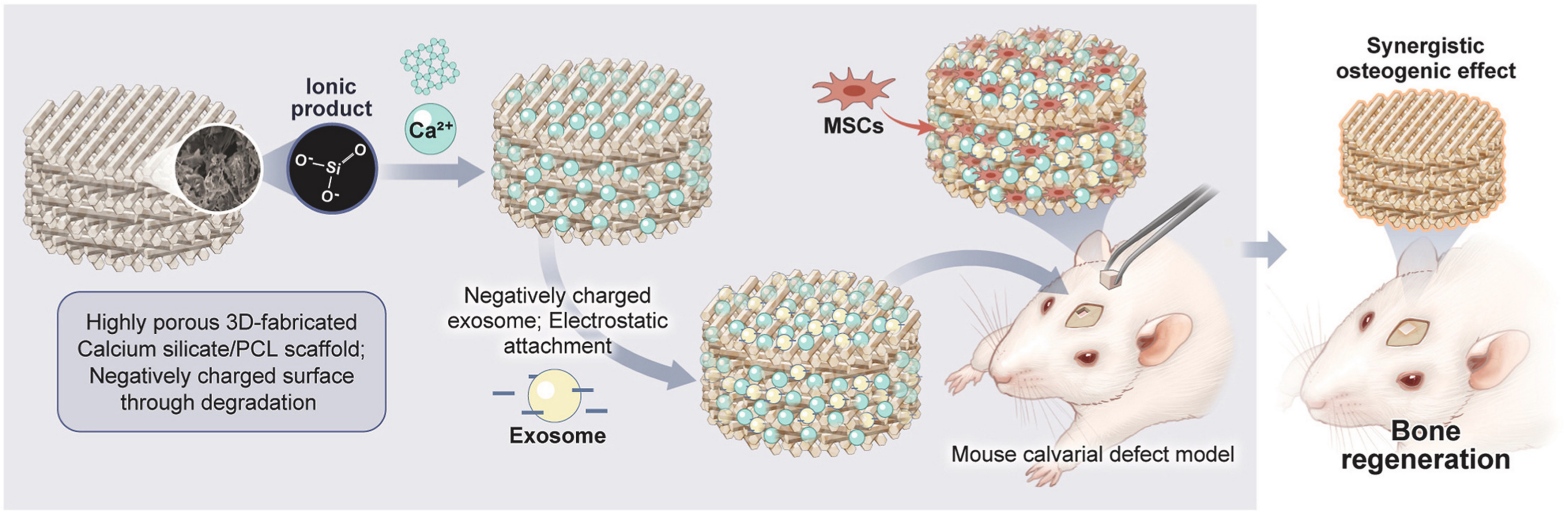
A 3D-fabricated Ca-Si scaffold is immersed in PCL, creating a composite base. This Ca-Si component acts as an affinity coating, significantly increasing the attachment of sEVs to the scaffold surface and enabling their sustained, controlled release. As a result, it overcomes limitations of rapid degradation. This synergistic platform promotes osteogenesis and stem cell recruitment for effective bone repair. Reprint with permission from Elsevier^[86]^. Ca-Si: Calcium silicate; PCL: polycaprolactone; sEVs: small extracellular vesicles; MSCs: mesenchymal stem cells.

The affinity immobilization strategy employs functional coatings to minimize burst release and achieve sustained delivery by enhancing sEV-material binding via defined intermolecular forces [Figure 3]. We modified injectable porous PLGA microspheres with polydopamine (PMS-PDA), which facilitates sEV loading and sustained release through electrostatic affinity with the sEVs membrane. Further enhancement was achieved by biomineralization to form a calcium phosphate coating (B/PDA@MS), which strengthens the interaction via Ca^2+^-phosphate coordination,enabling sustained sEV release for up to 21 days and promoting vascularized bone regeneration in rat calvarial defects^[51]^. Gandolfi *et al*. constructed calcium silicate (CaSi)/dicalcium phosphate dihydrate (DCPD)-modified PLA scaffolds for synergistic sEV delivery and osteoinduction^[100]^. Metal scaffolds coated with cationic polymers such as polylysine or polydopamine further improve sEVs loading through distinct mechanisms. Polylysine relies on electrostatic interactions with the anionic sEVs surface, while polydopamine forms covalent/non-covalent bonds via its catechol groups. For instance, polydopamine-modified titanium nanotubes incubated with sEVs at room temperature for 1-8 h enabled sustained uptake into BMSCs over 12 h^[101]^. Chitosan, polyethyleneimine (PEI), and other cationic molecules also show potential for delivery system modification by harnessing similar electrostatic principles^[102]^.

In our latest work, molecular dynamics simulations compared interactions between sEV membrane phospholipid bilayers and affinity coatings [PDA, tannic acid (TA), heparin, calcium phosphate compounds (CaP), PEI]. Interaction strengths followed: PDA < heparin < TA < CaP < PEI. Based on this, we selected PDA and CaP to modify PLGA porous micro-scaffolds (PLGAMS), yielding PDA@MS and biomineralized PDA@MS (B/PDA@MS) for sEV delivery. B/PDA@MS showed the highest loading efficiency (> 20 μg/mg scaffold) and optimal sEV release kinetics. Due to synergistic effects of biomineralization and sustained sEV release, sEV-loaded B/PDA@MS significantly promoted BMSC osteogenesis *in vitro* and bone regeneration in rat calvarial defects.

## HYDROGEL ENCAPSULATION

In contrast to the surface-dominated loading of scaffolds, hydrogels utilize their bulk hydrophilic network to encapsulate sEVs. This 3D entrapment offers distinct protection and release kinetics. Their highly tunable swelling capacity and biocompatibility make them indispensable for drug delivery and tissue repair. Encapsulating sEV within hydrogels serves a dual purpose: it protects them from rapid clearance and, upon direct application to the injury site, maintains a therapeutically effective local concentration.

### Direct incorporation strategy

**Figure 4:**
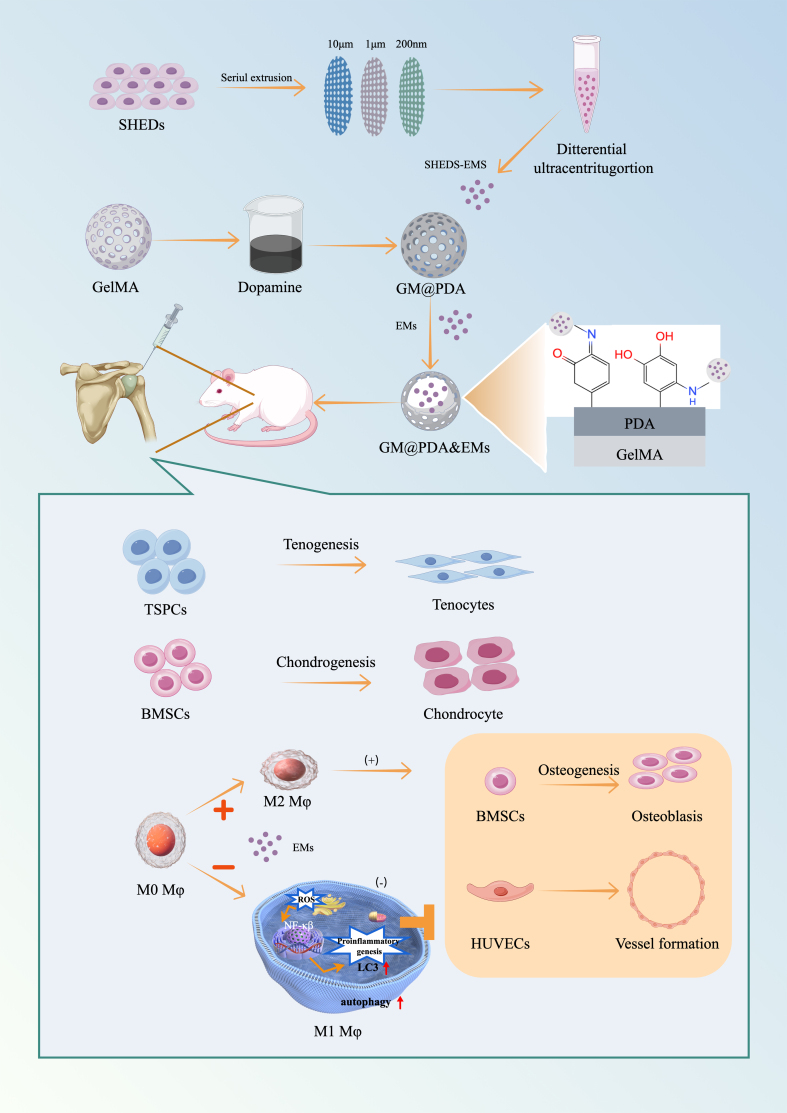
EMs derived from SHED are directly incorporated into a polydopamine-modified GelMA (GM@PDA) hydrogel network. This direct incorporation strategy ensures localized and sustained delivery of EMs at the tendon-bone interface. The released EMs modulate macrophage polarization toward the regenerative M2 phenotype via autophagy activation, suppress NF-κB-mediated inflammation, and concurrently enhance the tenogenic and chondrogenic differentiation of stem cells, thereby accelerating integrated repair. Reprint with permission from Elsevier^[108]^. EMs: Exosome-mimetics; SHED: stem cells from human exfoliated deciduous teeth; GelMA: gelatin methacryloyl; GM@PDA: GelMA hydrogel modified with polydopamine; NF-κB: nuclear factor kappa-light-chain-enhancer of activated B cells.

As a form of direct incorporation, integrating sEVs into hydrogels primarily relies on physical entrapment (steric hindrance). Common methods include premixing sEVs with hydrogel precursors prior to crosslinking or immersing swelling hydrogels in sEV solutions. Silk fibroin hydrogels were encapsulated with engineered sEVs derived from hypoxia-treated BMSCs to reduce nucleus pulposus cell senescence in both rat and human models, and ultimately delay intervertebral disc degeneration progression^[103]^. CS/ZnO-NPs (zinc oxide nanoparticles and chitosan hydrogel) immersed in HUVECs-derived sEVs solutions served as diabetic wound dressings in rats^[104]^. The adsorption and release kinetics of sEV depend on hydrogel crosslinking density, porosity, and environmental conditions (e.g., temperature, pH)^[105]^. Low porosity slows release, relying on hydrogel degradation/swelling. PEG macromers mixed with sEVs and intraperitoneally administered enabled sustained release over a month during biodegradation^[106]^. Hydrogel surface charge, hydrophilicity/hydrophobicity are key determinants of adsorption efficiency/stability. For example, phosphate groups on sEV membranes form reversible bonds with polyphenol groups (e.g., dopamine/TA), enabling sustained release for up to 14 days^[107]^. A PDA-modified GelMA hydrogel was constructed to enhance the loading efficiency of sEVs by electrostatic adsorption. SHED-sEVs-mimetics releases from PDA-modified GelMA hydrogel accelerates tendon-bone healing by modulating macrophage polarization and enhancing tissue regeneration [Figure 4]^[108]^.

### Affinity immobilization strategy

**Figure 5:**
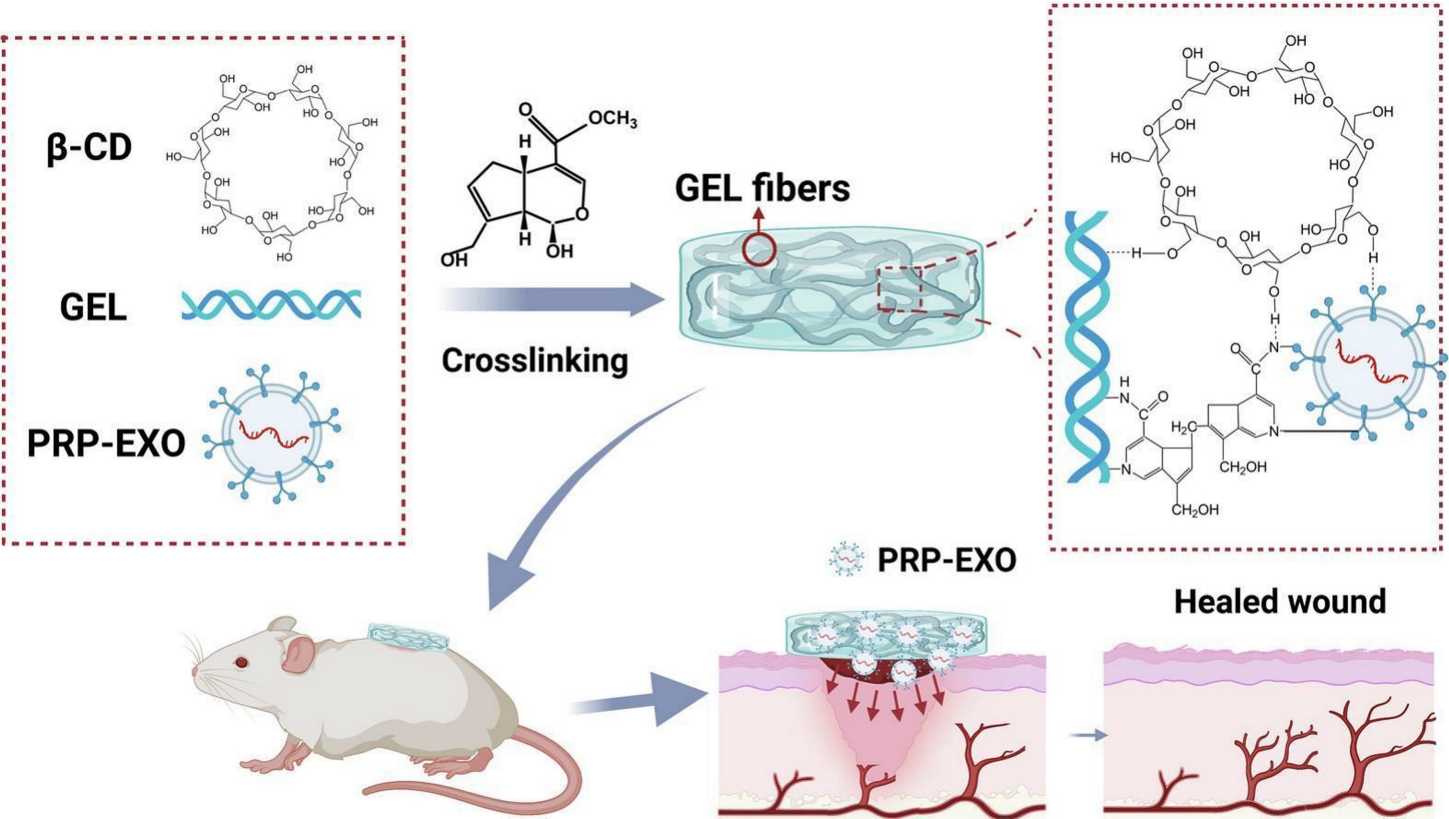
PRP-derived sEVs are integrated via an affinity immobilization strategy to prevent rapid clearance and enable sustained bioavailability. The immobilized sEVs directly counter high-glucose-induced pathologies by activating cellular autophagy and suppressing apoptosis in key skin cells. This targeted immobilization and release strategy, supported by the robust hydrogel scaffold, coordinately drives angiogenesis, collagen deposition, and re-epithelialization. Reprint with permission from Elsevier^[111]^. PRP: Platelet-rich plasma; sEVs: small extracellular vesicles; GEL: hydrogel; PRP-EXO: platelet-rich plasma-derived exosomes; β-CD: β-cyclodextrin.

Chemical immobilization represents an affinity immobilization strategy that achieves efficient sEVs retention by forming stable bonds (e.g., covalent crosslinks) between the hydrogel network and sEVs. For instance, carbodiimide/N-hydroxysuccinimide activates carboxyl groups to form amide bonds with amines, allowing sEVs surface proteins to covalently link to amino acid side chains within hydrogels^[109]^. Biocompatible crosslinkers such as genipin can stabilize sEVs-hydrogel integration while preserving bioactivity. Genipin, a natural compound from gardenia fruit, crosslinks amine-containing biomolecules (e.g., gelatin/polysaccharides) with lower toxicity than glutaraldehyde^[110]^. Platelet-rich plasma (PRP)-derived sEVs were loaded into genipin-crosslinked hydrogels. Scanning electron microscopy confirmed successful embedding [Figure 5]^[111]^.

Beyond these general chemical methods, a highly specific strategy involves the use of surface-engineered sEVs designed for high-affinity binding to hydrogel components. Fusion peptides represent a promising approach for engineering functional biomaterials, owing to their favorable attributes such as low molecular weight, low cytotoxicity, and high bioavailability. A key element in this strategy is the use of targeting peptides such as CP05, which specifically binds to the CD63 surface protein on sEVs. By integrating distinct functional modules, fusion peptides enable precise and stable immobilization of sEVs onto biomaterial substrates. This not only facilitates sustained sEVs release but also enhances the functional performance of the composite material.For instance, Zhang *et al*. designed a bifunctional fusion peptide consisting of a collagen-binding domain linked to CP05. This conjugate promoted efficient and uniform loading of sEVs onto collagen membranes by simultaneously targeting collagen and sEVs surface CD63, thereby improving both affinity and spatial distribution^[112]^. In a related study on diabetic wound repair, researchers employed a fusion peptide to anchor umbilical cord MSCs-derived sEVs onto a catechol-reinforced porcine small intestinal submucosa hydrogel. The CP05-mediated binding enabled prolonged sEVs retention and sustained release, which significantly enhanced granulation tissue formation and collagen deposition, accelerating wound closure^[113]^. Similarly, Ma *et al*. utilized a CP05-linked collagen-binding fusion peptide to immobilize BMSCs-derived sEVs on a reinforced hydrogel. This design enhanced sEVs retention and enabled controlled release, amplifying the osteogenic effect of sEVs and promoting healing in a skull defect model^[114]^.

## APPLICATIONS IN *IN SITU* TISSUE ENGINEERING

### Bone

Surgery, trauma, and systemic diseases often cause fractures. The subsequent bone repair process involves a complex healing cascade, including inflammatory cell recruitment, new blood vessel formation, and hematoma formation at the fracture site^[115]^. Given this context, sEVs have emerged as promising therapeutic agents to modulate and enhance this natural process. Lu *et al*. used tumor necrosis factor-α to precondition ADSCs and extracted their derived sEVs to induce bone tissue regeneration. Tumor necrosis factor-α-preconditioned adipose-derived stem cell-derived sEVs promoted the proliferation and differentiation of human primary osteoblasts and enhanced the expression of osteogenic-related genes^[116]^. Cui *et al*. collected mineralized osteoblast-derived sEVs, which promoted the differentiation of osteoblast precursors into mature osteoblasts *in vitro* by regulating osteoblast proliferation and mediating miRNA expression, thereby promoting new bone growth^[117]^. Combining human induced Pluripotent Stem Cells (hiPSC)-derived sEVs with β-TCP scaffolds effectively enhanced bone regeneration via the PI3K/AKT pathway. Simultaneously, in an immunocompetent rat osteochondral defect model, human induced pluripotent stem cell-derived sEVs promoted early cell infiltration and proliferation, induced synovial macrophage polarization, and exhibited anti-apoptotic activity to promote cartilage regeneration^[92]^. Skeletal stem cells (SSCs) derived fromthe infrapatellar fat pad exhibit high differentiation potential and robust chondrogenic capacity. Lou *et al*. used shape-adapted 3D-printed hydrogels for sustained release delivery of SSCs-derived sEVs to achieve simultaneous repair of cartilage and subchondral bone^[118]^.

Vascular formation is crucial for bone tissue regeneration. During bone repair, blood vessels deliver oxygen and nutrients to cells in the defect area^[119]^. SEVs derived from endothelial progenitor cells induced the formation of vascular-like structures *in vitro* and *in vivo* by activating endothelial Nitric Oxide Synthase (eNOS) and the PI3K/AKT pathway in HUVEC and human microvascular endothelial cells. Xie *et al*. prepared a novel sEV-functionalized scaffold using rat BMSC-derived sEVs combined with decalcified bone matrix, achieving enhanced pro-angiogenic and bone tissue regeneration effects. This sEV-functionalized scaffold demonstrated good neovascularization in nude mice subcutaneously, further verified by CD31 immunohistochemical staining^[120]^.

### Tendon

Tendon injury is a common orthopedic musculoskeletal system disease that causes unbearable pain and disability. Such injuries often occur during sports and daily activities^[121]^. The interface where tendons and ligaments attach to bone is the site of stress concentration in this region. Therefore, the anterior cruciate ligament and rotator cuff are the soft tissue structures most susceptible to injury from overuse^[122]^. In most cases, treating anterior cruciate ligament and rotator cuff injuries requires surgical tendon/ligament reconstruction, achieved by inserting tendon grafts into bone tunnels to establish tendon-to-bone healing. However, the recurrence rate of tearing after surgical intervention is high: the re-tear rate for repaired rotator cuffs is estimated to be as high as 94%, while the average failure rate for anterior cruciate ligament reconstruction is 11.7%^[123,124]^. To reduce the re-tear rate and achieve satisfactory surgical outcomes, various methods have been applied, such as different suture techniques, interference screws, tissue engineering, and growth factors, to promote tendon-bone healing in animal models and patients. However, efficacy is unsatisfactory due to the low number of tendon cells and poor vascularization at the injury site.

Tissue regeneration systems based on sEVs composite delivery materials have shown potential for repairing tendon injuries. Xu *et al*. found that using sodium alginate hydrogel to deliver mesenchymal stromal cell-derived sEVs accelerated tendon-bone healing and intra-articular graft remodeling after anterior cruciate ligament reconstruction^[125]^. Fu *et al*. loaded ADSC-derived sEVs into a hydrogel, which could induce tenocyte-derived stem cells to undergo osteogenic differentiation and promote adipogenesis *in vitro*, enhanced the expression of osteogenic differentiation-related genes, and improved the mechanical properties of the joint portion^[126]^. Huang *et al*. found that combining BMSC-derived sEVs with hydrogel to achieve sustained release of sEVs could promote fibrocartilage formation in a mouse tendon-bone construct model^[127]^.

### Skin

Skin wounds cause a series of local and even systemic physiological and pathological changes, imposing a heavy burden on patients and society. Wound healing is a highly continuous process for restoring skin barrier function, consisting of several complementary stages, including hemostasis, anti-inflammation, proliferation, and tissue remodeling^[128]^. During these processes, dynamic interactions exist among many different types of skin cells and immune cells, which play specific roles at particular stages to reshape the wound healing process^[129]^.

ADSC-, BMSC-, and HUVEC-derived sEVs are the most commonly studied types for wound healing. Zhou *et al*. compared the therapeutic effects of ADSCs and ADSC-derived sEVs on skin wounds using different administration methods. The results showed that ADSC-derived sEVs smearing and hADSC/hADSC-sEVs intravenous administration helped induce re-epithelialization at the defect, reduce scar width, and enhance angiogenesis and collagen synthesis^[130]^. Xiang *et al*. developed microneedle patch loaded with metal-organic framework (MOF) and ADSCs-derived sEVs for diabetic wound healing. The sEVs were assembled onto the MOF by electrostatic attraction. With the degradation of the microneedle patch, the MOF exerted an antimicrobial effect, and the released sEVs were taken up by the cells and enhanced tissue repair, the effectiveness of which was demonstrated in a diabetic wound healing model^[14]^. Tian *et al*. found ADSCs-derived sEVs accelerates diabetic foot ulcer wound healing and alleviates high glucose-induced fibroblast injury by regulating the expression of the Kelch-like ECH-associated protein 1/nuclear factor erythroid 2-related factor 2 (Keap1/Nrf2) axis^[131]^. In another study, a hydrogel platform was proposed to deliver sEVs secreted by human umbilical cord-MSCs-derived MSCs to promote systemic lupus erythematosus wound healing. *In vivo* imaging of small animals indicated that hUC-MSCs sEVs encapsulated within hydrogel could persist at the wound site for 9 days. The released sEVs were able to increase the levels of cell proliferation, migration and tube formation, with better granulation tissue formation effects and wound healing rates than controls^[132]^.

### Neural-related tissues

Spinal cord injury is a devastating trauma to the central nervous system, often resulting in loss of motor, sensory, and autonomic functions. A GelMA microneedle patch loaded with sEVs from three-dimensionally cultured MSCs can promote the transition of microglia from the M1 to M2 phenotype in the post-spinal cord injury microenvironment, reducing neuroinflammatory responses and showing significant neuroprotective effects^[133]^. Luo *et al*. constructed a hydrogel-based sEV delivery system. M2 macrophage-derived sEVs could enhance the angiogenic activity of spinal cord microvascular endothelial cells *in vitro*. The sustained release of sEVs mediated by the hydrogel significantly promoted vascular regeneration and functional recovery at the injury site in mice. Proteomic studies found that this superior pro-repair effect originated from the high expression of ubiquitin thioesterase within M2 macrophage-derived sEVs, which increased protein expression levels by inhibiting β-catenin ubiquitination, activated Wnt/β-catenin signaling, and promoted cell proliferation^[134]^.

Traumatic brain injury refers to brain dysfunction caused by external force to the head, leading to coma, impaired consciousness, intracranial hemorrhage, cerebral edema, and even epileptic seizures^[135,136]^. Vascular system damage, activation of microglial inflammation, and excessive release of reactive oxygen species during trauma are major obstacles to patient recovery. Li *et al*. designed a hybrid hydrogel based on bioactive antioxidants for the sustained release and delivery of SHED-derived sEVs to treat traumatic brain injury. The multifunctional hydrogel, besides having antioxidant capabilities, also possesses thermosensitive, injectable, and self-healing properties, enabling long-term sustained release of cellular sEVs over 21 days. *In vivo*, an antioxidant hydrogel integrated with SHED-derived sEVs eliminated ROS, promoted microglial M2 polarization, rescued motor function, induced cortical regeneration, and reduced cerebral edema in traumatic rats^[137]^. Liu *et al*. constructed a hyaluronic acid-collagen hydrogel that simulated the brain matrix environment. Through the sustained release of BMSC-derived sEVs, neural stem cells were recruited and their differentiation fate was modulated. The sEVs promoted differentiation into neuronal cells and oligodendrocytes. While simultaneously inhibiting astrocyte differentiation, as evidenced by downregulated glial fibrillary acidic protein (GFAP) expression. This differentiation shift reduced glial scar formation, synergistically promoting vascularization and neurogenesis in the lesion area, leading to axonal regeneration, myelination, synapse formation, and brain remodeling for neural function recovery^[138]^.

## CHALLENGES AND OPPORTUNITIES

Over the past decade, the synergistic combination of sEVs and delivery systems has shown immense potential in the field of tissue engineering and regenerative medicine. In the future, through the integration of materials science and engineering technology, advanced delivery systems that endow sEVs with precise spatiotemporal-controlled release capabilities represent possible development directions. One key research direction includes modulating the interactions between the biomaterial carrier and sEVs to achieve on-demand control over release kinetics, thereby maintaining therapeutic sEV concentrations that align with the distinct timelines of various tissue repair processes. Another direction involves engineering delivery systems to transition sEV release from passive diffusion to actively responsive mechanisms (e.g., temperature, pH, or enzyme), allowing for precise spatiotemporal control and maximizing sEV enrichment and efficacy at the injury site.

### Long-term sustained release

During tissue regeneration, transient bursts of bio-signals inducing regeneration can easily lead to repair stagnation or localized fibrosis. There is an urgent need to develop sustainable delivery systems to extend the therapeutic window. Hydrogel delivery carriers exemplify sEV release rate depends on crosslinking density and molecular weight of crosslinkers. Higher density reduces porosity, extending release; lower molecular weight increases density, also slowing release. Kwak *et al*. controlled crosslinking density and porosity to regulate the degradation time of polyethylene glycol hydrogel between 6 and 27 days, and further regulated the release efficiency of macrophage-derived sEVs to maximize their therapeutic effect in skin wound healing^[139]^. In contrast to conventional delivery reliant on passive diffusion, an alternative “on-demand” strategy can be achieved by employing surface modifications that strongly bind to sEVs. Su *et al*. modified the polycaprolactone (PCL) fibers with PEI, which has a strong electrostatic adsorption with sEVs, showing a minimal initial detachment of the sEVs residuals. The sEVs immobilized on the scaffold would not be released unless they came into direct contact with the cell and then were taken up by the cell, realizing a dynamic “on-demand” process^[85]^.

### Responsive release

Environmentally responsive release of sEVs within hydrogels is an intelligent drug delivery strategy that allows sEVs to be released in response to specific biological or physicochemical environmental changes. This method employs specially designed hydrogel materials to ensure the effective release of sEVs at the correct time and target location. Hydrogels used for sEV delivery can respond to environmental changes such as temperature, photothermal effects, pH, and enzymes.

Temperature-responsive release in hydrogel-sEV systems typically uses synthetic materials such as polyethylene glycol or poly(N-isopropylacrylamide) (PNIPAM) to prepare injectable gels with controllable biochemical properties. Zhang *et al*. incorporated platelet-rich plasma-derived sEVs into a thermosensitive hydrogel. At 25 °C, 90% of the sEVs were released in 4 days; at 37 °C, the sustained release effect was extended to 28 days. Continuous release within 28 days promoted the proliferation and migration of mouse bone marrow MSCs and chondrocytes *in vitro*, simultaneously enhanced the chondrogenic differentiation of mouse bone marrow MSCs, and inhibited inflammation-induced chondrocyte apoptosis; cartilage protection in osteoarthritis was achieved *in vivo*^[140]^. The design of photothermal-responsive hydrogel systems for drug delivery mainly utilizes the material’s ability to absorb near-infrared light and convert it into heat, thereby triggering drug release. Metal nanoparticles, carbon nanomaterials, and polymer nanocomposites are often selected as efficient photothermal converters. Ma *et al*. constructed a thermoresponsive hybrid hydrogel based on graphene oxide, which periodically released sEVs under near-infrared radiation, synergistically promoting the proliferation and migration of Schwann cells^[141]^. The pH-responsive mechanism for sEV release in hydrogel systems exploits the phenomenon where hydrogels change states such as swelling, shrinking, or dissolving under specific pH conditions. Key polymers such as polyacrylic acid, polymethacrylic acid, and polyhistidine have been used in such studies. The design of pH-sensitive hydrogels requires ensuring the stability of sEVs during pH changes and customizing suitable sustained-release triggering conditions according to the pH of the target body site. Wang developed an injectable, self-healing, and antibacterial peptide hydrogel with pH-responsive capability. In a pH = 5.5 environment, the release rate of sEVs within 21 days was 95%; whereas in a pH = 7.5 environment, the sustained release amount of sEVs decreased to 85%. This pH-responsive sustained release effect of sEVs significantly improved the proliferation, migration, and angiogenic capacity of HUVEC and promoted chronic wound healing *in vivo*^[142]^.

## CONCLUSION

SEVs delivery systems provide dynamic and precise solutions for *in situ* tissue repair by integrating biological functions with materials engineering. Future research on the extraction and engineering of sEVs will be very important, aiming to improve the efficiency and quality of sEVs by selecting appropriate sources and engineering strategies. Simultaneously, in-depth understanding of the characteristics of different materials is crucial to customize and modify materials to adapt to different application environments and requirements, achieving more precise and controlled sEVs release. Ultimately, simplifying preparation processes and enhancing stability are critical steps to surmount the translational barriers and facilitate the clinical adoption of these innovative sEV-based regenerative strategies.

## DECLARATIONS

### Availability of data and materials

The data used to support the findings of this study are available from the corresponding author upon request.

### AI and AI-assisted tools statement

During the preparation of this manuscript, the AI tool DeepSeek was used solely for language editing. The tool did not influence the study design, data collection, analysis, interpretation, or the scientific content of the work. All authors take full responsibility for the accuracy, integrity, and final content of the manuscript.

### Copyright

© The Author(s) 2026.
